# Flexible sensor matrix film-based wearable plantar pressure force measurement and analysis system

**DOI:** 10.1371/journal.pone.0237090

**Published:** 2020-08-07

**Authors:** Shumi Zhao, Rong Liu, Chengwei Fei, Abdul Wasy Zia, Lingxiao Jing

**Affiliations:** Institute of Textiles and Clothing, The Hong Kong Polytechnic University, Hung Hom, Kowloon, Hong Kong SAR, China; University of California Davis, UNITED STATES

## Abstract

Plantar pressure force data derived from gait and posture are commonly used as health indicators for foot diagnosis, injury prevention, and rehabilitation. This study developed a wearable plantar pressure force measurement and analysis (WPPFMA) system based on a flexible sensor matrix film to monitor plantar pressure force in real time. The developed system comprised a flexible sensor matrix film embedded in the insole of the shoe, a wearable data acquisition (DAQ) device with a Bluetooth module, and dedicated software with an intuitive graphical user interface for displaying the plantar pressure force data from receivers by using a terminal unit (laptop or smart-phone). The flexible sensor matrix film integrated 16 piezoresistive cell sensors to detect pressure force at different anatomical zones of the plantar and under different body positions. The signals from the flexible sensor matrix film were collected using the DAQ module embedded in the shoe and transmitted to the receivers through Bluetooth. The real-time display and analysis software can monitor, visualize, and record the detailed plantar pressure force data, such as average pressure force, maximum pressure force, and pressure force distributions and variations over time. The outcomes of the trials in which the system was worn revealed the applicability of the developed WPPFMA system for monitoring plantar pressure force under static and dynamic wearing conditions. The plantar pressure force data derived from this system provide valuable insights for personal foot care, gait analysis, and clinical diagnosis.

## 1. Introduction

Plantar pressure force information is commonly applied to determine and analyze various foot disorders and impairments [1, 2]. Continuous efforts have been deployed for improving the effectiveness and reliability of the plantar pressure and force measurement. Woodburn et al. [3] designed a commercialized in-shoe F-Scan system to test plantar force and pressure. Shu et al. [4] reported a six-textile-fabric sensor-based in-shoe analysis system for plantar pressure detection. Gerlach et al. [5] printed the composite pressure sensor for plantar pressure monitoring. Rajala et al. [6] applied piezoelectric polymer films with evaporated copper electrodes to fabricate lightweight and cost-effective matrix film sensors. Lou et al. [7] developed multilayer graphene films by using polyester textiles to detect plantar pressure. Tan et al. [8] introduced a low-cost smart insole with a sandwiched structure into which a carbon-based piezoresistive material was inserted between two layers of electrodes for real-time plantar pressure measurement. Moreover, a wireless sensing system that is integrated into a shoe has been used in gait analysis to collect plantar pressure force data [9, 10]. However, constructing a real-time foot measurement and analysis system remains challenging. It was found that the developed in-shoe F-scan system had calibration errors and unfavorable durability [3], and a single sensor integrated into the shoes produced unstable measurements because of a lack of systematic assembly of the sensors into a film for smooth signal collection. Therefore, further development of an effective wearable plantar pressure force measurement and analysis system is essentially valuable to enhance the measurement performance in practical applications.

At present, some wireless-wearable plantar pressure force systems have been developed and applied for acquiring personal health information [11–14]. For example, Arafsha et al. [11] used a wireless cyber-physical system to monitor, visualize, and record gait data by using conventional IEEE 802.11WiFi. By integrating a waist belt and shoes, Silva et al. [12] designed a remote care-taking system to measure plantar pressure under a pre-defined posture and activity by calculating gait parameters. Deng et al. [13] developed a facile plantar pressure mapping system through piezoelectric nanogenerators and a mobile display. However, the developed WiFi system was not user friendly because of the large embedded battery [14, 15]. For data collection during long-period of activity, a plantar pressure force measurement system that is compact, lightweight, comfortable, and low-cost is more suitable for users’ everyday wear. In addition, the graphical user interface (GUI) is an essential element for the real-time displaying plantar pressure force profiles. Currently, much effort has been made on the design and development of hardware testing systems, whereas insufficient attention has been paid to user-friendly human-machine interaction in the measurement of plantar pressure force.

This study designed a flexible sensor matrix film-based wearable plantar pressure force measurement and analysis (WPPFMA) system for personalized foot health and activity monitoring. The developed system consisted of a flexible sensor matrix film that was placed into shoe, a wearable data acquisition (DAQ) device with a Bluetooth module and display receiver (laptop or smartphone), and dedicated software. The developed flexible sensor matrix film comprised 16 piezoresistive sensor cells to detect the plantar pressure force magnitudes and distributions across the 16 different anatomic zones of the foot plantar. The signals from the flexible sensor matrix film were collected using a stable DAQ module embedded in the insole and were transmitted to the receiver through a Bluetooth module. A user-friendly GUI software was engineered to monitor, visualize, and record plantar pressure force data in real time, including pressure force spatial distributions and average and maximum pressures force. Trials that involves wearing the system were conducted to validate the efficiency of the developed flexible sensor matrix film-based WPPFMA system.

## 2. Materials and methods

### 2.1 Fabrication of the flexible sensor matrix film

The real-time measurement of plantar pressure force requires a sensor to be placed in the insole of a shoe for accurate measurement [13, 16]. The optimal layouts of the insole sensors were selected to match the morphologies of insoles and shoes to fit the individual’s foot plantar shape [17, 18]. The three-dimensional plantar shape influences the contact area of the plantar sensor. Some small zones cannot be fully covered by the plantar sensor layout. Therefore, the average pressure force of an insole surface detected by the insole sensor is commonly used to indicate plantar pressure force, a technique that is considered to yield acceptable precision relative to adjacent areas [19, 20]. In this study, a time-integral mean plantar pressure force derived from practical testing was used to determine the pressure force value of each sensor cell.

**Fig 1:**
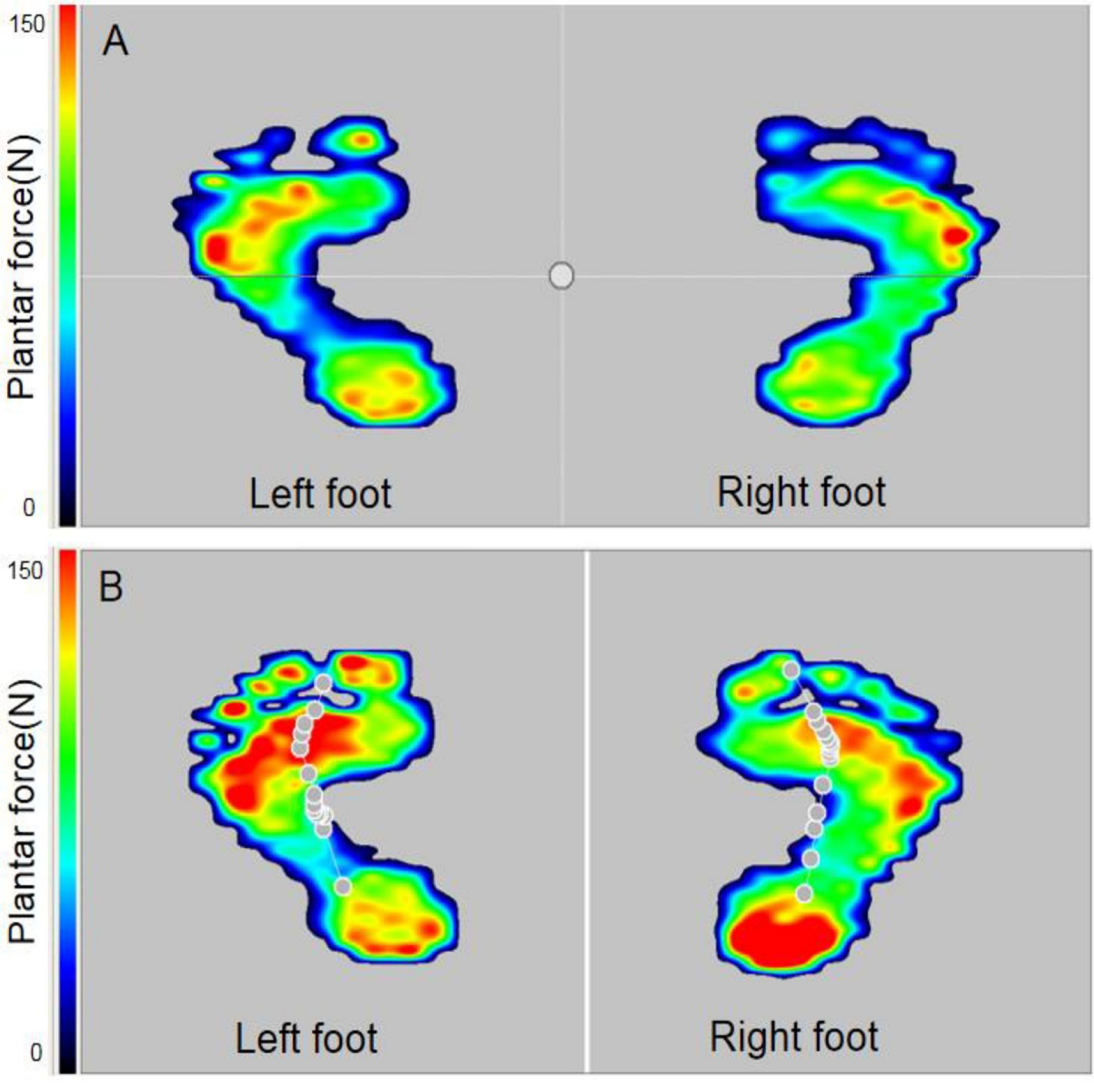
Diagrams of (A) static and (B) dynamic pressures of the barefoot tested using Gaitview system.

The plantar pressure force test was performed using a Gaitview® AFA-50 system (alFOOTs, Seoul, South Korea), and a subject walked on the pressure force sensing mat barefoot. Fig 1 illustrates the tested plantar pressures force in static and dynamic conditions. Plantar pressure force profiles were determined by the morphologies and anatomic structures of the tested subjects’ feet [21, 22]. In general, the metatarsal toe area at the forefoot, lateral arch at the midfoot, and heel at the rearfoot had higher pressures force [20, 21]. Peek plantar pressure force commonly appeared at the bony prominence portions, such as the distal, middle and proximal phalanxes and calcaneus.

**Fig 2:**
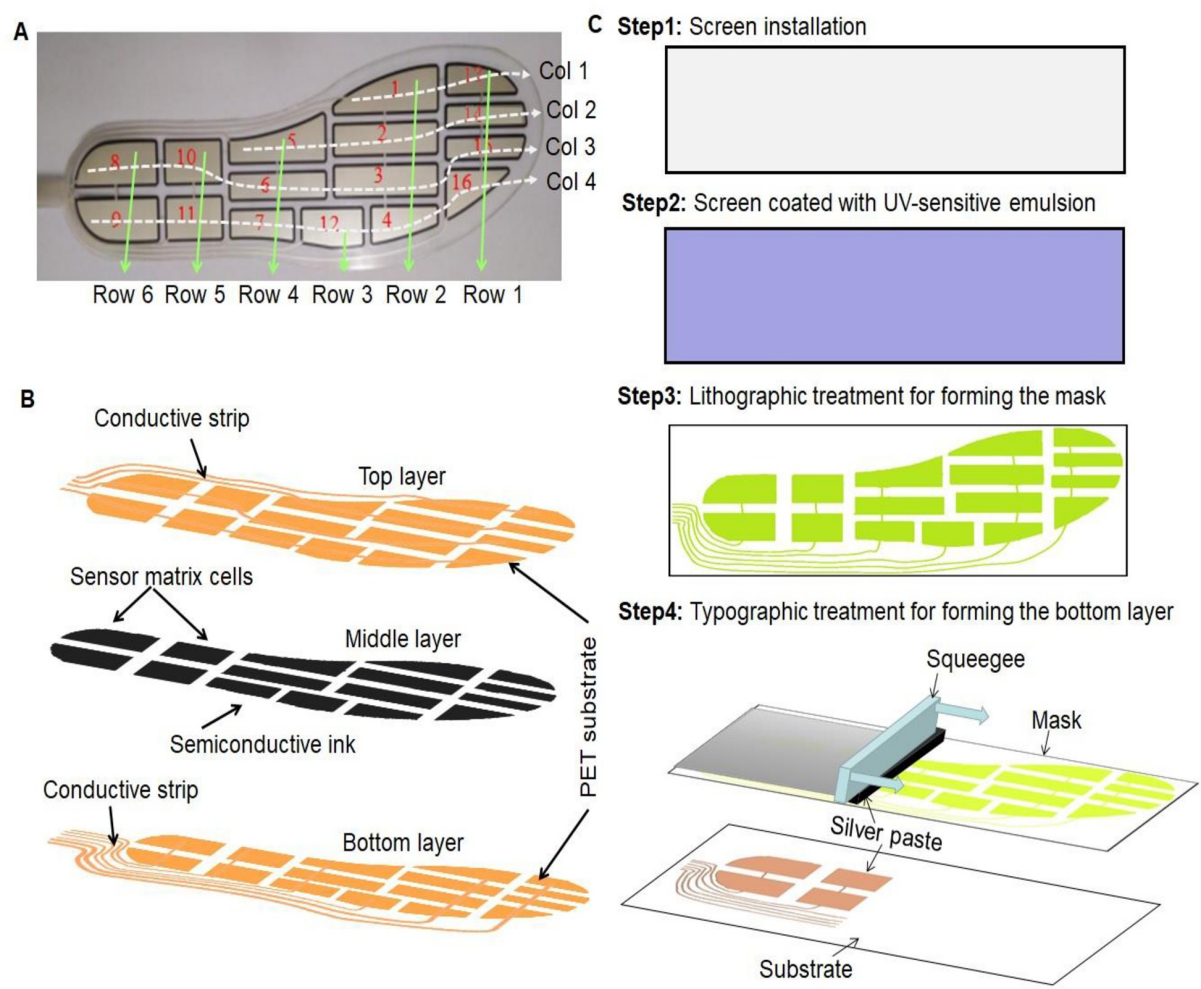
Flexible sensor matrix film with 16 piezoresistive sensors for plantar pressure force measurement, (A) flexible sensor matrix film (col: column), (B) the topologic structure of the flexible sensor matrix film, and (C) fabrication of the flexible sensor matrix film.

To detect plantar pressure force distributions, a new flexible sensor matrix film was developed as shown in Fig 2 A, that involved 16 piezoresistive sensor cells that distributed across four columns and six rows. The divisions of the 16 sensing zones were identified based on the foot anatomical structure and biomechanical profiles of the human feet during static and dynamic activities [20, 22]. These 16 zones included the aforementioned plantar zones, which are sensitive to external plantar forces. In general, the four sensor cells (1–4) were set at the metatarsals zones, four (5–7,12) at the plantar arch, four (8–11) at the calcaneus zones, and four (13–16) at the phalanges zones.

The developed flexible sensor matrix film was a sandwich structure that comprised three main layers (Fig 2B). These layers included the top and bottom layers made of polyethylene terephthalate (PET) materials and a middle layer of nano-force-sensitive semiconductive ink. These layers were formed using advanced screen printing technologies [23, 24]. Fig 2C depicts the fabrication process of the flexible sensor matrix film.

First, the stainless steel or polyester-woven screen was mounted onto a metal frame under appropriate tension; Second, the screen was coated using ultraviolet-sensitive emulsion materials and processed using lithography treatment to form the masks with 16 pressure sensing areas and strips [23, 24]. Two groups of conductive strips were placed at the top and bottom layer masks, respectively, which linked all the predetermined 16 sensor cells. The strip placement is detailed as follows. The first group of strips were longitudinally set along the top layer mask, in which the first strip (column 1) connected with the 1st and 13rd sensing areas, and the second strip (column 2) connected with the 2th, 5th and 14th sensing areas. The third strip (column 3) connected with the 8th, 10th, 6th, 3rd, and 15th sensory areas, and the fourth strip (column 4) connected with the 9th,11th,7th,12th, 4th, and 16th sensory areas. The second group of strips were horizontally set along the bottom layer mask, where the first strip (row 1) connected with the 13th,14th,15th, and 16th sensing areas, and the second strip (row 2) connected with the 1st, 2th, 3rd, and 4th sensing areas. The third strip (row 3) connected with the 12th sensing area, and the fourth strip (row 4) connected with 5th, 6th, and 7th sensing areas. The fifth strip (row 5) connected with the 10th and 11th sensing areas, and the sixth strip (row 6) connected with the 8th and 9th sensing areas. Third, silver paste was poured onto the top surface of the bottom layer mask, and a squeegee was used to move the paste from one end to another end of the treated mask under an appropriate pressing force. Thus, the conductive strips were printed onto the PET-based bottom layer. Fourth, through the same method, the nano-force-sensitive semiconductive ink was poured onto the top surface of the middle layer mask. Then, a squeegee was moved under appropriate pressure to facilitate the penetration and stickiness of the semiconductive ink onto the PET-based bottom layer. Fifth, the procedure was repeated. The silver paste was poured onto the surface of the top layer mask, and a squeegee was moved to make the silver paste penetrate and stick to the PET-based top layer. Finally, the bottom, middle, and top layers were laminated together to form a flexible sensor matrix film. The sensor resistance range of the fabricated film was from approximate 0.5 kΩ to ∞ in its detection of plantar pressure force (material mode: RX-ES40-16P, Shenzhen Rouxi Technology Co, Shenzhen, China).

Applied pressure force is negatively related to equivalent resistance [7, 8]. Thus, when an external force is applied, the resistance on the fabricated pressure force sensor decreases. The generated output voltage signals from the flexible sensor matrix film were collected and transmitted by the DAQ system to the software used for data processing and real-time display of the detected plantar pressure force.

### 2.2 Data acquisition unit

**Fig 3:**
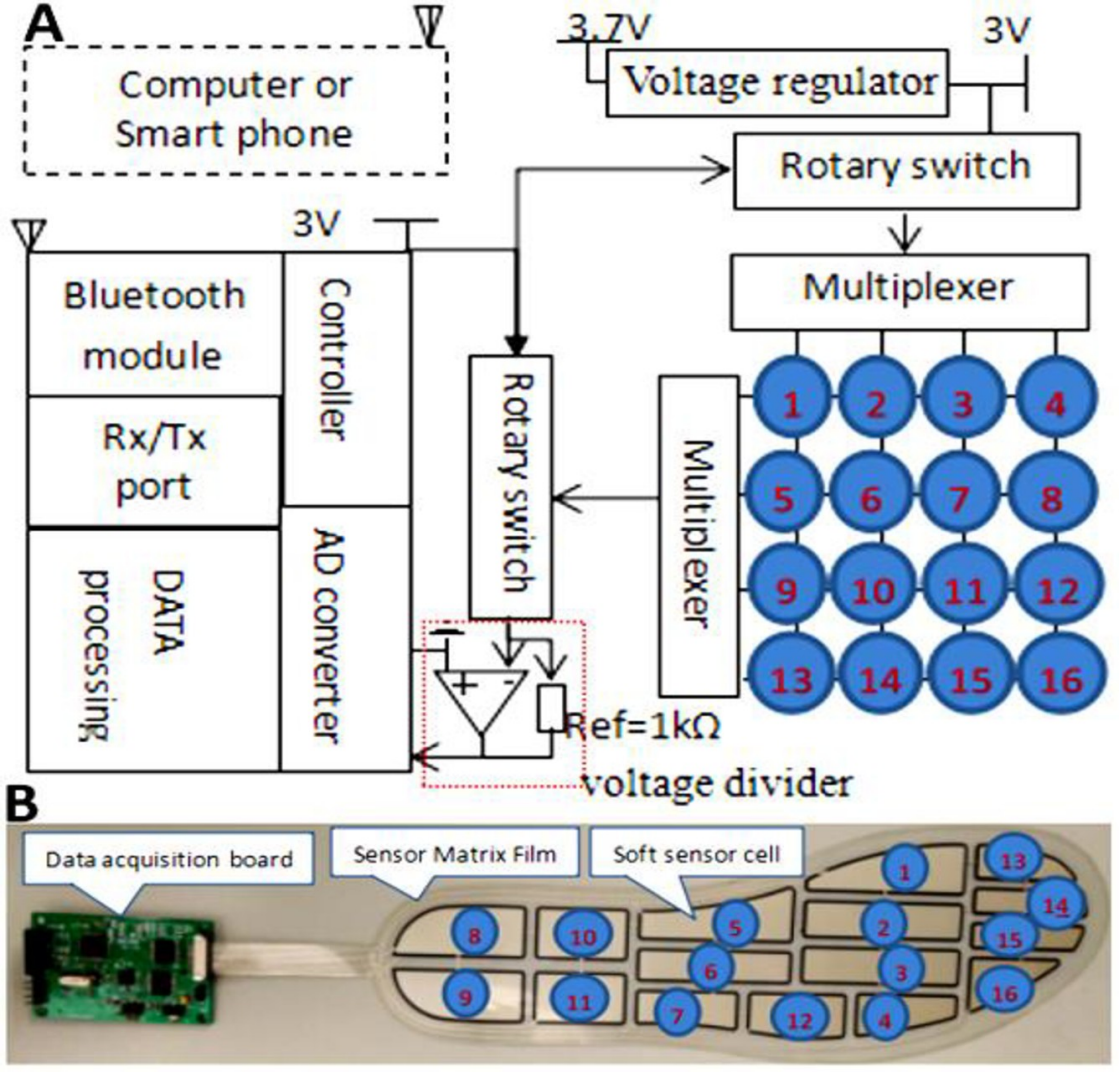
(A) Data acquisition model and (B) data acquisition module of DAQ system.

The DAQ unit is the core element of the WPPFMA system (Fig 3). It comprises a Bluetooth module, set of Rx/Tx ports, voltage regulator, data processing unit, controller, A/D converter, and rotary switches. The voltage regulator was used to convert 3.7 V of Li-ion battery to 3 V as a system voltage. Each cell of the soft matrix film was selected by two rotary switches for data collection. The voltage signals of the cells were extracted from the voltage divider and then sent to the A/D converter channels installed in the microcontroller STM32 (STMicroelectronics, Geneva, Switzerland), where the voltages were transformed into force values by using a 12-bit A/D converter. Through the serial (Rx/Tx) port, the force values were sent to the Bluetooth module, and then the Bluetooth antenna wirelessly transmitted the force values to a remote receiver (e.g., smart-phone or laptop).

To calculate the voltage data, the flexible sensor matrix film was connected to the reference resistor (*R*_*ref*_ = 1 kÙ). The experience value was set according to sensor resistance (0.5 kÙ → ∞) by using a rotary switch and connecting wires. The input analog voltage (*A*_*input*_) can be calculated using the following voltage division equation:

$$A_{input}=R_{ref}*V_{cc}/\left(R_{ref}+R_{sensor}\right)$$ (1)

where *V*_*cc*_ is the system voltage, and *R*_*sensor*_ is the resistance of the cell sensors.

The digital output of voltage (*D*_*output*_) can be calculated using Eq (2):

$$D_{output}=2^{m}*A_{input}/V_{ref}$$ (2)

where *V*_*ref*_ is the input full-scale voltage, and *m* is the bit length of *D*_*output*_. The *V*_*ref*_ of the A/D converter was connected to *V*_*cc*_.

*V*_*ref*_ = *V*_*cc*_, Eq (2) can be derived to be Eq (3),

$$D_{output}=2^{m}*A_{input}/V_{ref}=\left[2^{m}*V_{cc}*R_{ref}\left(R_{ref}+R_{sensor}\right)/V_{cc}\right]=2^{m}*R_{ref}\left(R_{ref}+R_{sensor}\right)$$ (3)

**Fig 4:**
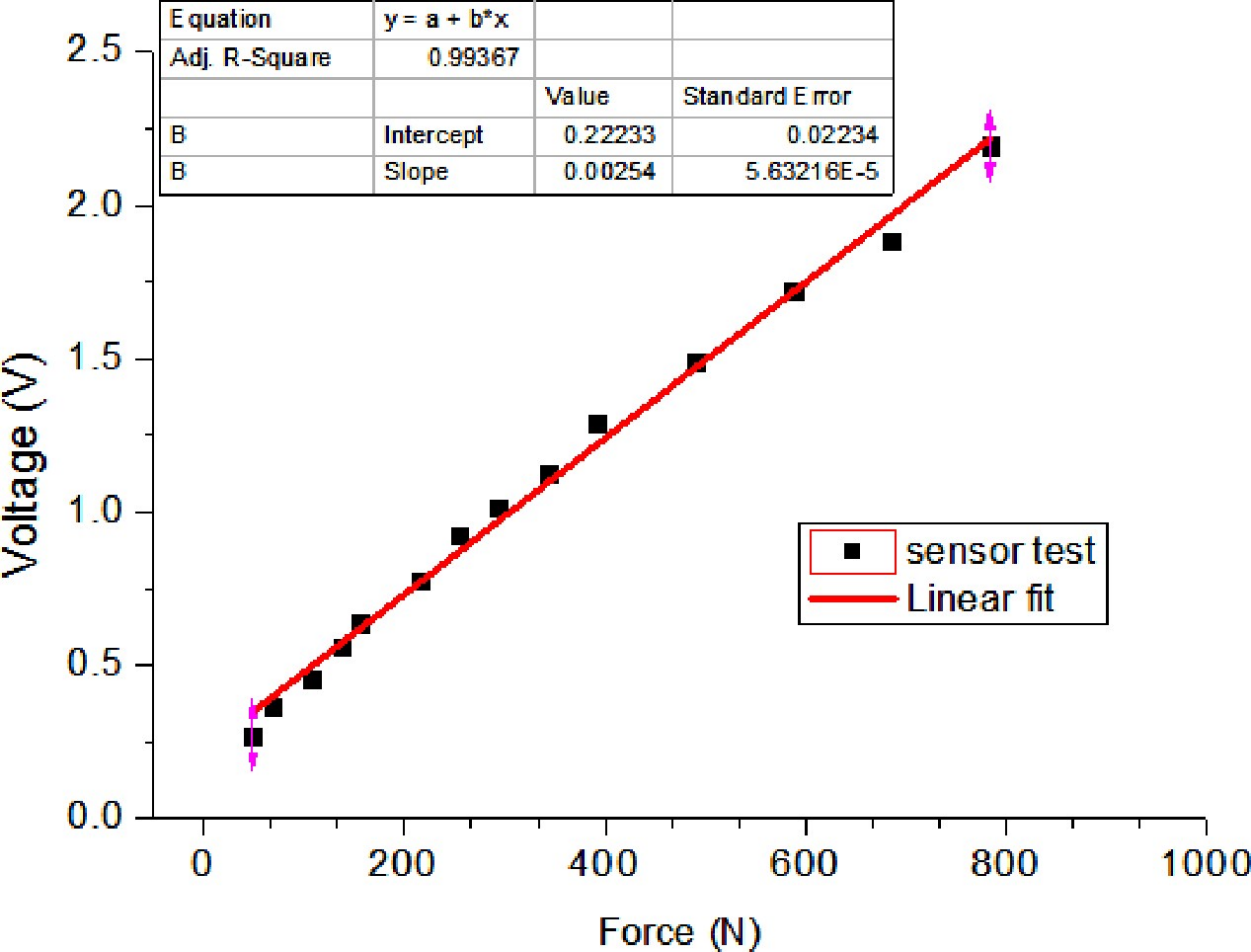
Relationship between the cell voltage and the external applied force for the 11th cell of the flexible sensor matrix film.

The relationship between the force values and the sensor resistance was determined, and *D*_*output*_ is independent of *V*_*cc*_. Each cell of the flexible sensor matrix film was calibrated using counterweights with different weight levels according to standard testing procedures [25, 26]. Fig 4 illustrates a representative calibration process for cell 11. The voltages of the sensor cell proportionally increased with increasing external force applied. The developed DAQ system could detect the applied force from 0.98 to 980 N toward each cell of the flexible sensor matrix film.

### 2.3 Development of data monitoring and analysis software

In this study, a dedicated GUI was developed and installed in a smart-phone to record, visualize, and analyze the collected pressure data. The details on the data processing and GUI development are presented as follows.

#### 2.3.1 Pressure data processing

The developed software system was installed in the terminal unit (smart-phone or laptop), which consisted of three stacked components: the physical driver interface, data preprocessing module, and data post-processing module. The software was developed by applying multi-threading technology to perform real-time data computing. Six main threads were designed in a software program. The details are as follows. The first device driver thread was used to handle asynchronous communication by using the Bluetooth serial port profile. The device driver synchronized the collected pressure signal data before forwarding them to the client programs through interconnect sockets. The second thread performed data pre-processing, which involved de-noising, filtering, and calibrating the collected pressure signals and estimating the sensor baseline. The acquired pressure data from the different sensing zones were transmitted to the corresponding service programs and were processed in the data post-processing module. The remaining four threads received the pre-processed data and performed two local and two remote services, respectively. The two local services included pressure data calibration, update, and save. The two remote services included pressure data curve drawing and dynamic display. During data reception and processing, data packages were read from the virtual serial port data buffer (physical driver interface) by using the device driver thread and the data were then extracted from the data packages, and transformed into pressure values by using the following voltage–force curves of the sensor cells based on the methods detailed in Section 2.2 for pressure calibration (Fig 4).

#### 2.3.2 Graphical user interface

**Fig 5:**
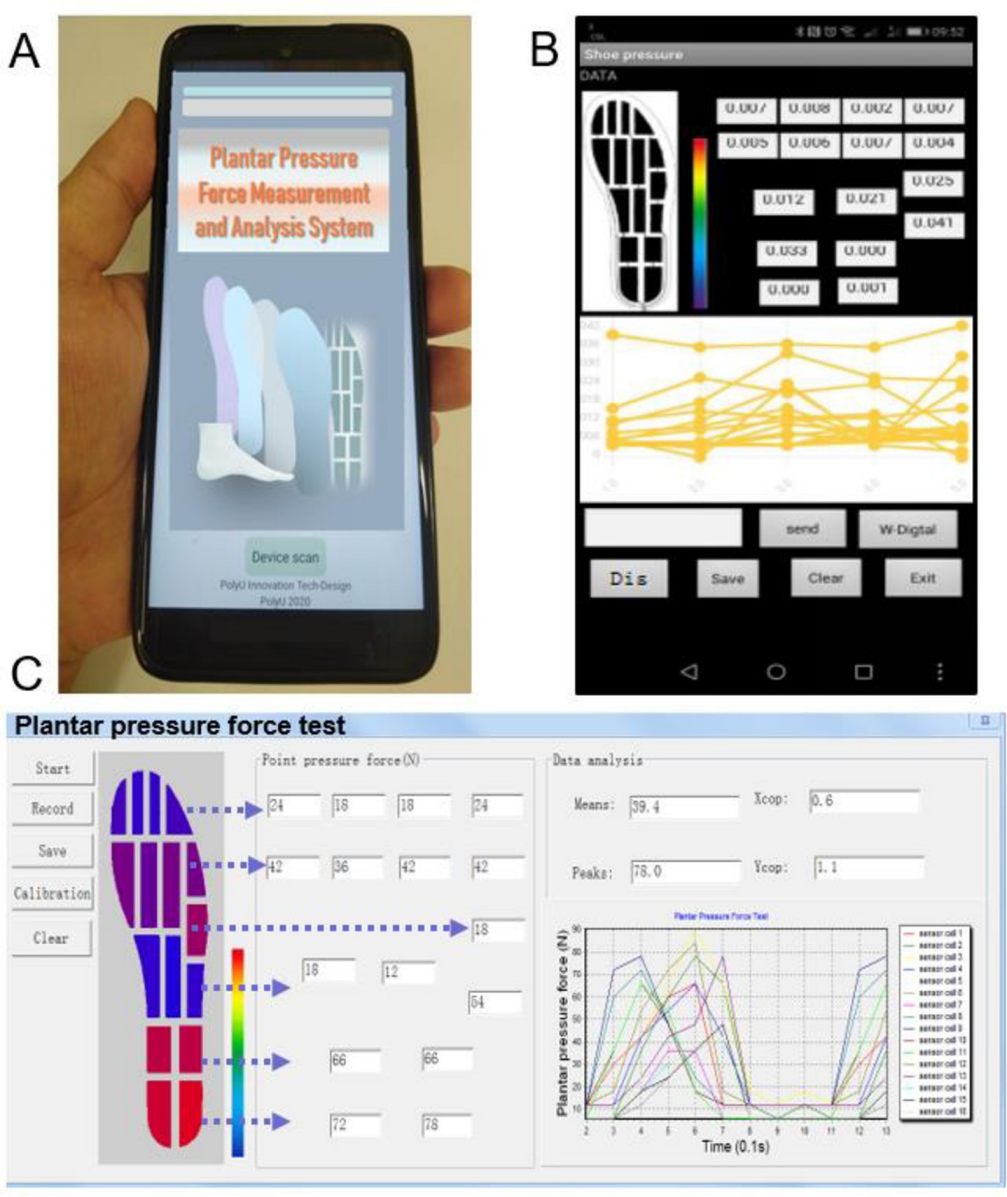
The developed GUI for the different terminal unit (laptop or and smart-phone). (A) Application preface, (B) Data display interface of a smart phone, and (C) Interface of the laptop.

Fig 5A and 5B present a preface and data monitoring panel in the mobile version of the user-friendly GUI that this study developed. In the GUI, the plantar pressure force and pressure force-time curves of each sensor cell are observed and recorded in real-time. The tabs for user commands are set for convenient use (Fig 5B) in the GUI. Fig 5C presents an overview of the developed GUI for desktop (and laptop) systems. The desktop GUI is more versatile relative to its mobile counterpart in the computing and mapping of the distributions of mean pressure force, peak pressure force, and center of pressure force on the plantar in real time. The plantar pressure force can be calculated using Eqs (4)–(6),

$$Mean=\frac{1}{n}\sum_{i=1}^{n}p_{i}$$ (4)

$$Peak=Max(p_{1},\ldots ,p_{i},\ldots ,p_{n})$$ (5)

$$\left\{ \begin{array}{l} X_{cop}=\frac{\sum_{i=1}^{n}X_{i}P_{i}}{\sum_{i=1}^{n}P_{i}}\\ Y_{cop}=\frac{\sum_{i=1}^{n}Y_{i}P_{i}}{\sum_{i=1}^{n}P_{i}} \end{array}\right.$$ (6)

where *p* is the plantar pressure force, *n* denotes the total number of sensors; *i* denotes the *i*th sensor, and *X* and *Y* are the coordinates of the whole foot area. Fig 5C displays the colour-graded pressure force map presented in the GUI that allows the user to quickly review the pressure force distributions in different sensing zones of the plantar. The middle section of the GUI presents specific values for each sensor cell, and the right side of the GUI presents the curves of plantar pressure force against time, as detected by the sensor cells. The developed system can monitor plantar pressure force during everyday activities, such as running, walking, and sitting. Furthermore, users can apply the developed WPPMFA system to review historical data to understand plantar pressure force variations, in a given period, for analyzing foot biomechanical features or diagnosing disorders.

To validate the applicability of the developed WPPFMA system, the wear trials were designed and carried out to detect the plantar pressure force under various body activities. The protocol of the trial was approved by the Human Subjects Ethics Sub-committee of the Hong Kong Polytechnic University (HSEARS20180503001) where the participant provided their written consent.

## 3 Results and discussion

The experimental outcomes of the newly developed sensor-film based plantar pressure force measurement system are illustrated as follows. One subject with a 46-kg body weight and a European 40 shoe size [27] was recruited to perform plantar pressure force measurements under static and dynamic conditions, including standing upright on one foot or both feet, sitting, natural walking, and other daily actives—such as running, jumping, and climbing stairs.

### 3.1 Static plantar pressure force measurement

Table 1 depicts the plantar pressure force data detected by the developed sensor matrix-based WPPFMA system when the subject was standing on one foot and both feet. A higher plantar pressure force was distributed at the heel area (cells 8, 9, 10, and 11) and the metatarsal area (cells 1, 2, 3, and 4), which is consistent with results in the literature obtained from tests on normal human feet using a Gaitview® AFA-50 system [28, 29]. Variations in feet anatomical structures, such as pes planus (low medial longitudinal arch) or pes cavus (high medial longitudinal arch), are associate with increased body weight or lower limb (particularly, foot) injuries—including obesity, medial tibial stress syndrome, and patellofemoral pain [30, 31]. The profiles of foot plantar pressure force are influenced by the foot anatomical structure and body position. Users can learn about healthy conditions of the foot by observing plantar pressure force magnitudes and distributions and take appropriate measures to care or nurse their feet timely.

**Table 1:** Static plantar pressure force under natural standing position.

Sensor Cell	Standing on both feet (N)		Standing on the single-foot (N)	
	Right foot	Left foot	Right foot	Left foot
**1**	15.1	06.8	28.5	18.8
**2**	23.2	19.0	35.8	48.2
**3**	34.2	29.9	41.6	47.4
**4**	04.8	09.1	36.8	16.3
**5**	01.7	02.5	10.7	04.1
**6**	01.6	04.8	07.0	03.7
**7**	01.9	02.2	01.0	06.0
**8**	23.4	25.0	77.8	119.9
**9**	38.7	43.1	58.4	84.9
**10**	41.7	31.1	37.9	49.3
**11**	25.5	25.4	36.5	32.9
**12**	03.1	07.2	22.8	07.3
**13**	04.9	03.6	14.8	04.4
**14**	03.2	03.8	07.4	04.5
**15**	04.7	04.1	13.2	04.8
**16**	04.2	05.4	20.5	04.2
**Total**	231.9	223.0	450.7	456.7

As shown in Table 1, the total pressure forces (455N) of the right (232N) and left feet (223N) were similar to those while standing on one foot for the right foot (450.7N) or left foot (456.7N) when the subject was in a natural upright standing position. This result reflected the sensitivity of the developed flexible sensor matrix film in practical application. The testing error values were less than 10%, which indicated favorable accuracy in the developed system.

### 3.2 Dynamic plantar pressure force measurement

**Fig 6:**
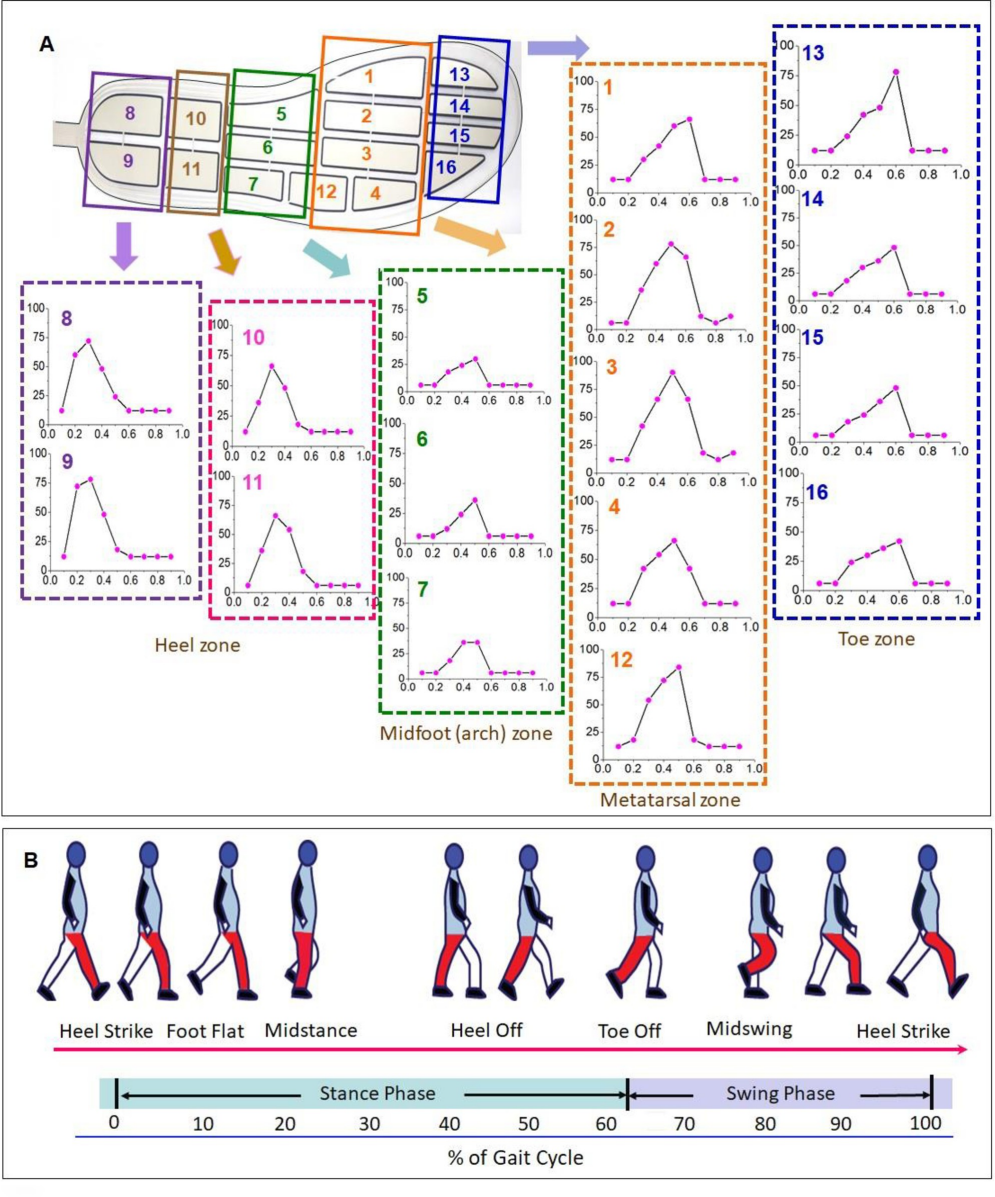
Plantar pressure force variations of the tested 16 sensor cells under a normal gait cycle (X-axis: Time (s), Y-axis: Force (N). Along the x-axis, 0s-0.2s indicate the stage of the heel-strike; 0.2s-0.4s indicate the stages of the foot-flat, 0.4s-0.5s indicate the stage of the midstance, 0.5s-0.6s indicates the stage of the heel-off, 0.6s-0.65s indicates the stage of the toe-off, and 0.6s-1s indicate the stage of the midswing).

Fig 6 displays the variation profiles of the plantar pressure force detected by the 16 sensor cells during a dynamic gait cycle, including the stages of heel strike, foot flat, midstance, heel off, and toe off midswing. The plantar pressure force at the heel and metatarsal areas were generally higher than those of the positions of the tarsals and phalanges. The 8th and 9th sensor cells located at the internal and lateral heel area dramatically increased when the heel struck the ground. The plantar pressure force detected by the 13–16th sensor cells were located at the 1st and 2nd cells. Metatarsals areas steeply increased during the toe-off stage. As the subject moved forward, the plantar pressure force of the remaining sensor cells responded sequentially from the heel toward the toes, and the peak pressure force moved from the heel (the 8th–11st cells) to the midfoot (5th–7th and 12th cells) and then to the metatarsals (1st–4th cells) and the toes (13rd–16th cells) over the gait time. When the heel lifted off the ground, the pressure force detected by the 8th sensor cell returned to its values at the initial stage. When the toes pushed the ground for forward propulsion, instant peak pressure force occurred, which was detected by the 13th sensor at the forefoot. When the entire foot was off the ground during the swing phase, the plantar pressure force on all sensor cells returned to their initial conditions.

Gait condition influences the balance and stabilization of the body during movement [32–34]. A gait cycle is commonly divided into the stance and swing phases. The plantar pressure force in a gait cycle was measured using the developed flexible sensor matrix film-based WPPFMA system and a commercial testing system (Gaitview® AFA-50 system), respectively.

**Fig 7:**
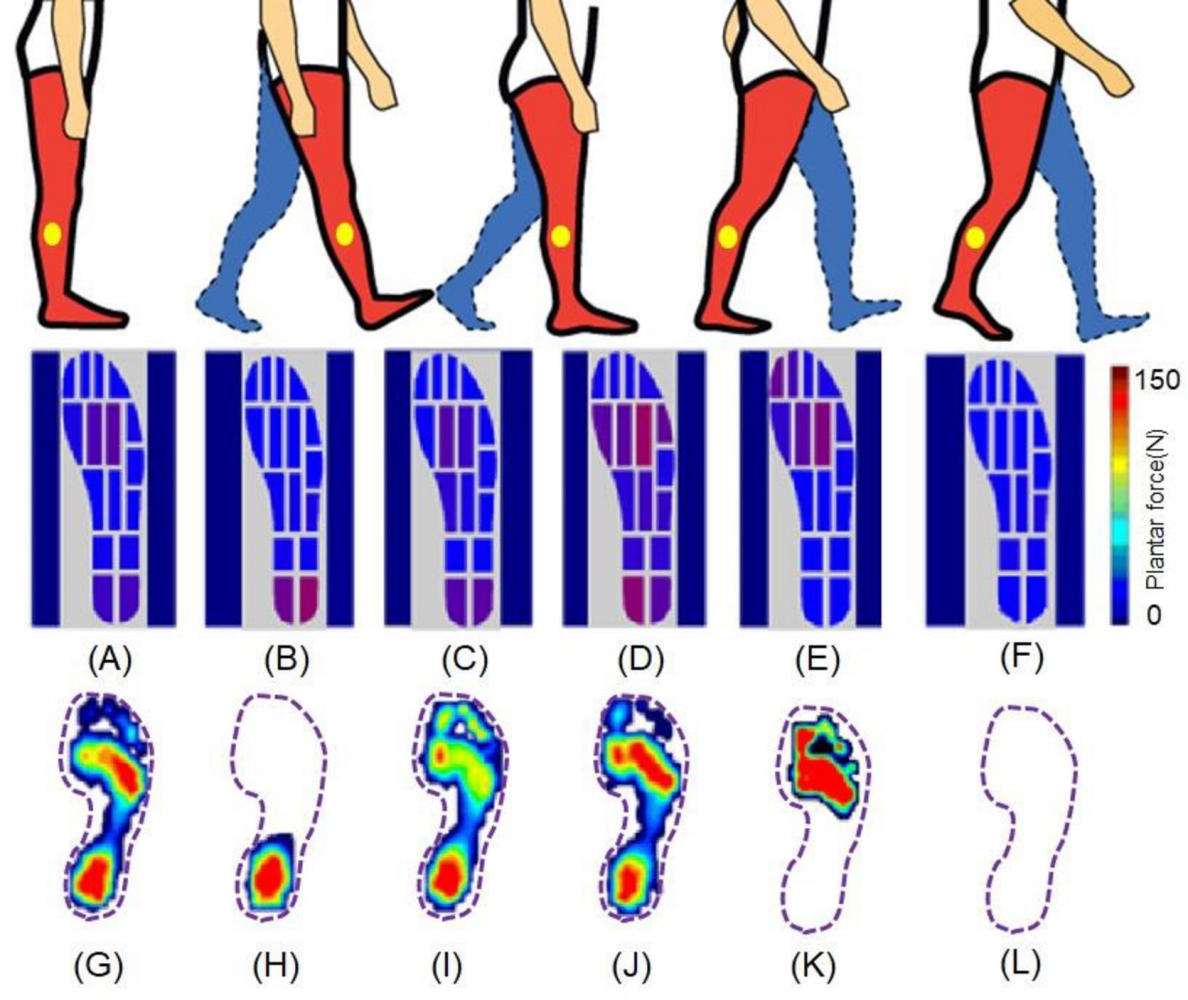
Comparison on the plantar pressure force distributions of the right foot under different gait phases measured by the developed sensor-film based WPPMA system (A-F), and the commercial pressure force measurement system (Gaitview® AFA-50 system) (G-I). (A: stance, B: heel-strike, C: foot-flat, D: midstance, and E: heel-off, and F: midswing).

Plantar pressure force mapping was graded on a color scale ranging from dark blue (low force of 10N) to dark red (high force of 150N; Fig 7A–7F). The plantar pressure force varied by changes in gait phases. At the static standing position, the body weight was evenly distributed over the whole gait plantar (Fig 7A). When moving from the rearfoot landing on the ground to the forefoot landing off the ground in the swing phase, the body weight was transferred correspondingly from the rearfoot to the forefoot, which was dynamically monitored, visualized, and recorded by the developed WPPFMA system, as displayed in Fig 7. The developed software simultaneously performed numerical computation to determine key parameter such as average pressure force and maximum pressure force, in addition to mapping the curves of pressure force and its variation against time. The results indicated that the detected plantar pressure force distributions (Fig 7A–7F) at different stages of the gait cycle were consistent with the outcomes derived by the commercial pressure force measurement device (Fig 7G–7L).

The heel strike was initially on the posterolateral site of the heel, where the plantar pressures force at the heel reached its peak value at approximately 25% of the stance phase. The pressure force center was intense in the initial heel strike stage, which was then followed by a rapid force transfer forward. At this moment, the midfoot pressure force was generally displayed as lower. Because the center of gravity was transferred from the hindfoot to the forefoot in midstance, the center of the plantar pressure force passed through the midfoot, which resulted in a raised midfoot plantar pressure force. The contact time between the heel and midfoot on the ground occupied approximately 50% of the stance phase, and the peak plantar pressure force occurred at the 80% of the stance phase. Studies have reported that a higher pressure force is commonly distributed at the first, second, and third metatarsal heads of the hallux valgus feet [35, 36], whereas a significantly higher pressure force occurs at the hallux. The third and fourth metatarsal heads of the hallux limitus feet indicate a more lateral forefoot loading. Dynamic plantar pressure force data derived from the developed WPPFMA system helped determine plantar pressure force distributions, thus allowing for the analysis of feet anatomical structures for personal foot care, clinical diagnosis, and daily health monitoring.

### 3.3 Case study of plantar pressure force measurement for routine activities

**Fig 8:**
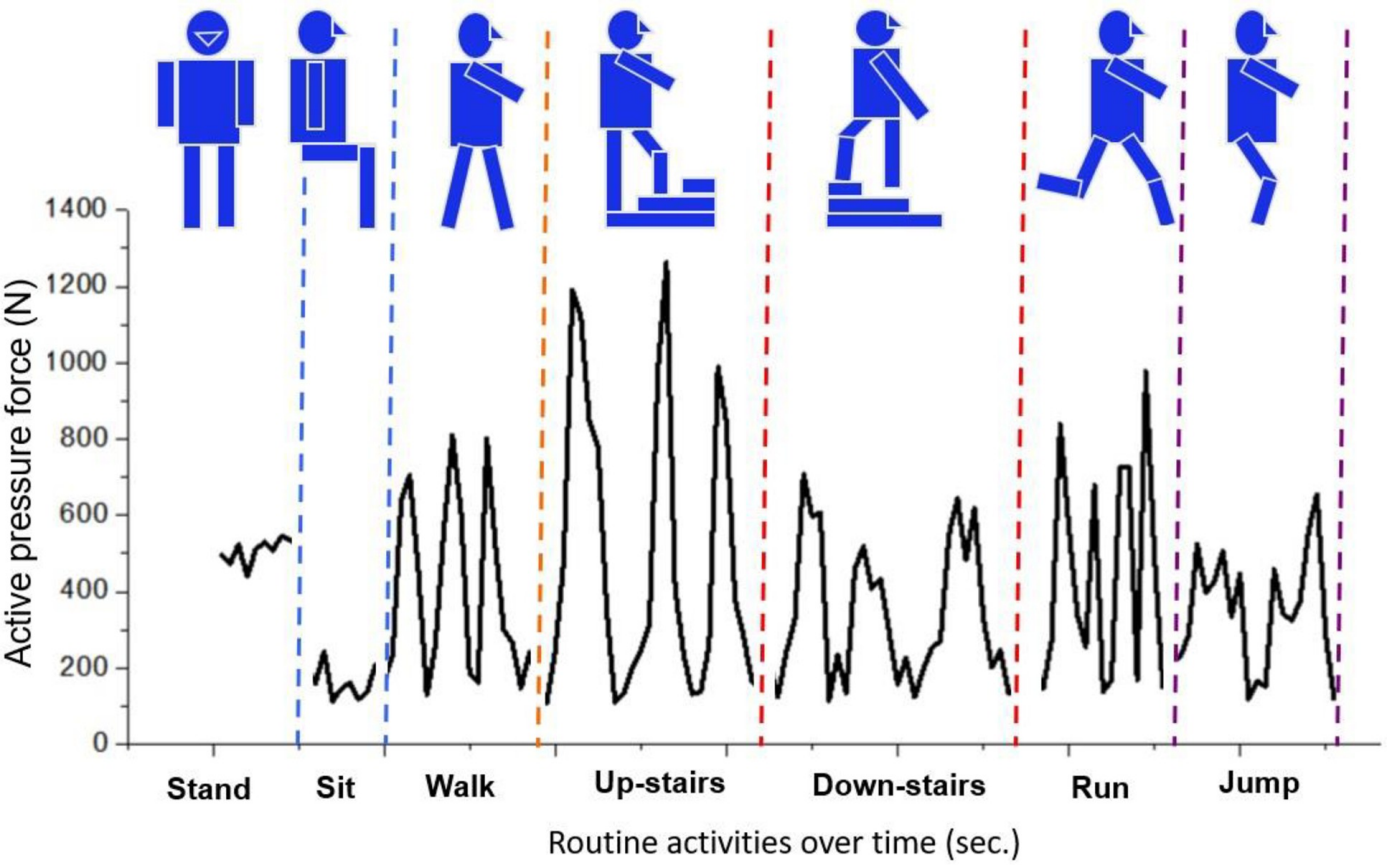
The real-time plantar pressure force mapping of the routine activities measured by the developed sensor film-based WPPFMA.

To analyze the efficiency and sensitivity of the developed flexible sensor matrix film-based WPPFMA system, a case study was performed in a trial in which the system was worn. The recruited subject (weight: 76kg; height: 170cm; body mass index: 26.3 kg/m^2^) performed a series of activities, including standing, sitting, walking, ascending stairs, descending stairs, running, and jumping, to simulate everyday physical activity. Fig 8 illustrates the plantar pressure force and variations detected by the developed WPPFMA system.

The plantar pressure force output was stable at the standing and sitting positions. A periodical change in the plantar pressure force occurred during dynamic activities. A 50% higher plantar pressure force occurred when the subject ascended stairs than that when descending stairs. Similarly, a higher frequency of the pressure force cycle occurred when the subject ran than when the subject walked. Although the foot plantar fully contacted the floor when walking and jumping, their plantar pressure force profiles were extremely different. More steep peaks in the plantar pressure force profile occurred in the jumping position than those in the standing and walking positions. The results reliably validated the applicability of the developed sensor film-based system for plantar pressure force detection under static and dynamic conditions.

## 4. Conclusion

An in-shoe WPPFMA system based on a flexible sensor matrix film was developed in this study to measure the magnitudes and distributions of plantar pressure force among 16 anatomical sites. A stable DAQ system that was connected to the insole-plantar sensor transmitted the acquired pressure force signal data to a receiver by using a Bluetooth device. Dedicated software with a user-friendly GUI was developed, which was installed in the terminal unit (a laptop or smartphone) to monitor, visualize, and record the detailed plantar force data in real time. The developed software computed the collected pressure force signal and converted the data into readable pressure forces, including the average pressure force, maximum pressure force, and their dynamic variations with time. In addition, the developed system allows for the visualization and analysis of historical data for users to track their health. The study results revealed the applicability of the developed system on plantar pressure force measurement under static and dynamic conditions by comparing the experimental data with those generated by a commercial measurement system. The developed WPPFMA system presented satisfying measurement accuracy in real-time and was capable of visualizing a color-graded map of the 16 nodes located at different anatomical zones on the whole sensing sole. The case study indicated that the developed system was reliable and robust in its response when measuring plantar pressure force during daily routine activities. Therefore, the plantar pressure force data derived from this system can aid the efficient performance of personal foot care for gait analysis and clinical diagnosis in diverse wearing conditions.

## Supporting information

S1 File (DOCX)
