# Supplementary material for: Flexible sensor matrix film-based wearable plantar pressure force measurement and analysis system
Source: PLoS One. 2020 Aug 7;15(8):e0237090. doi: 10.1371/journal.pone.0237090 (PMC7413492; doi:10.1371/journal.pone.0237090)
Supplement: S1 File — (DOCX) [file pone.0237090.s001.docx]

**PONE-D-20-02663R1 Supporting information file**

**Raw data of Figure 4**

| Force(N) | Voltage(V) |
| --- | --- |
| 68.6 | 0.36842 |
| 107.8 | 0.45763 |
| 137.2 | 0.56098 |
| 156.8 | 0.63780 |
| 215.6 | 0.77778 |
| 254.8 | 0.92797 |
| 294 | 1.01500 |
| 343 | 1.12561 |
| 392 | 1.29165 |
| 490 | 1.49143 |
| 588 | 1.72340 |
| 686 | 1.88792 |
| 784 | 2.19329 |

**Raw data of Figure 6**

| time(s) | cell1(N) | cell2(N) | cell3(N) | cell4(N) | cell5(N) | cell6(N) | cell7(N) | cell8(N) | cell9(N) | cell10(N) | cell11(N) | cell12(N) | cell13(N) | cell14(N) | cell15(N) | cell16(N) |
| --- | --- | --- | --- | --- | --- | --- | --- | --- | --- | --- | --- | --- | --- | --- | --- | --- |
| 0.1 | 12 | 6 | 12 | 12 | 6 | 6 | 6 | 12 | 12 | 12 | 6 | 12 | 12 | 6 | 6 | 6 |
| 0.2 | 30 | 6 | 12 | 12 | 6 | 6 | 6 | 60 | 72 | 36 | 36 | 18 | 12 | 6 | 6 | 6 |
| 0.3 | 42 | 36 | 42 | 42 | 18 | 12 | 18 | 72 | 78 | 66 | 66 | 54 | 24 | 18 | 18 | 24 |
| 0.4 | 60 | 60 | 66 | 54 | 24 | 24 | 36 | 48 | 48 | 48 | 54 | 72 | 42 | 30 | 24 | 30 |
| 0.5 | 66 | 78 | 90 | 66 | 30 | 36 | 36 | 24 | 18 | 18 | 18 | 84 | 48 | 36 | 36 | 36 |
| 0.6 | 12 | 66 | 66 | 42 | 6 | 6 | 6 | 12 | 12 | 12 | 6 | 18 | 78 | 48 | 48 | 42 |
| 0.7 | 12 | 12 | 18 | 12 | 6 | 6 | 6 | 12 | 12 | 12 | 6 | 12 | 12 | 6 | 6 | 6 |
| 0.8 | 12 | 6 | 12 | 12 | 6 | 6 | 6 | 12 | 12 | 12 | 6 | 12 | 12 | 6 | 6 | 6 |
| 0.9 | 12 | 12 | 18 | 12 | 6 | 6 | 6 | 12 | 12 | 12 | 6 | 12 | 12 | 6 | 6 | 6 |

**Raw data of Figure 8**

| Stand (N) | Sit (N) | Walk (N) | Up-stairs (N) | Down-stairs (N) | Run (N) | Jump (N) |
| --- | --- | --- | --- | --- | --- | --- |
| 495.6 | 198.2 | 232.6 | 106.8 | 123.4 | 145.0 | 227.4 |
| 472.6 | 159.0 | 637.2 | 245.8 | 241.8 | 270.4 | 286.4 |
| 522.8 | 242.6 | 704.0 | 482.2 | 325.8 | 838.6 | 524.6 |
| 440.6 | 113.0 | 457.6 | 1190.8 | 707.4 | 569.6 | 396.4 |
| 509.0 | 144.8 | 128.0 | 1119.4 | 597.4 | 338.0 | 426.6 |
| 530.2 | 161.2 | 260.4 | 845.0 | 605.8 | 255.2 | 504.6 |
| 507.8 | 117.4 | 568.2 | 781.4 | 113.8 | 677.8 | 333.8 |
| 545.4 | 135.0 | 810.4 | 352.4 | 234.2 | 137.2 | 447.0 |
| 535.4 | 206.0 | 605.4 | 108.6 | 132.6 | 167.8 | 118.2 |
| 525.0 | 164.6 | 183.4 | 132.6 | 457.6 | 726.8 | 166.2 |
|  |  | 161.4 | 197.4 | 518.4 | 727.6 | 151.4 |
|  |  | 801.4 | 245.0 | 408.0 | 168.6 | 458.4 |
|  |  | 526.4 | 312.2 | 432.4 | 977.0 | 343.0 |
|  |  | 298.0 | 978.0 | 295.6 | 494.6 | 324.6 |
|  |  | 266.2 | 1263 | 156.0 | 150.0 | 370.0 |
|  |  | 147.0 | 412.2 | 226.0 |  | 557.4 |
|  |  | 241.4 | 236.0 | 123.2 |  | 654.6 |
|  |  |  | 131.2 | 194.6 |  | 286.2 |
|  |  |  | 137.8 | 251.6 |  | 119.4 |
|  |  |  | 263.4 | 270.0 |  |  |
|  |  |  | 988.2 | 548.4 |  |  |
|  |  |  | 844.8 | 643.6 |  |  |
|  |  |  | 377.8 | 483.2 |  |  |
|  |  |  | 283.6 | 619.4 |  |  |
|  |  |  | 162.2 | 334.8 |  |  |
|  |  |  | 140.6 | 200.8 |  |  |
|  |  |  |  | 246.0 |  |  |
|  |  |  |  | 132.2 |  |  |

**Raw data of Table 1**

| Standing on both feet | | Standing on the single-foot | |
| --- | --- | --- | --- |
| Right foot (N) | Left foot (N) | Right foot (N) | Left foot (N) |
| 15.1 | 6.8 | 28.5 | 18.8 |
| 23.2 | 19.0 | 35.8 | 48.2 |
| 34.2 | 29.9 | 41.6 | 47.4 |
| 4.8 | 9.1 | 36.8 | 16.3 |
| 1.7 | 2.5 | 10.7 | 4.1 |
| 1.6 | 4.8 | 7.0 | 3.7 |
| 1.9 | 2.2 | 1.0 | 6.0 |
| 23.4 | 25.0 | 77.8 | 119.9 |
| 38.7 | 43.1 | 58.4 | 84.9 |
| 41.7 | 31.1 | 37.9 | 49.3 |
| 25.5 | 25.4 | 36.5 | 32.9 |
| 3.1 | 7.2 | 22.8 | 7.3 |
| 4.9 | 3.6 | 14.8 | 4.4 |
| 3.2 | 3.8 | 7.4 | 4.5 |
| 4.7 | 4.1 | 13.2 | 4.8 |
| 4.2 | 5.4 | 20.5 | 4.2 |
